# Optimizing Patient Care: A Systematic Review of Multidisciplinary Approaches for SLE Management

**DOI:** 10.3390/jcm12124059

**Published:** 2023-06-15

**Authors:** Giorgio Galoppini, Antonio Marangoni, Francesca Cirilli, Francesca Ruffilli, Carlo Garaffoni, Marcello Govoni, Carlo Alberto Scirè, Ettore Silvagni, Alessandra Bortoluzzi

**Affiliations:** 1 Rheumatology Unit, Department of Medical Sciences, Università degli Studi di Ferrara, Azienda Ospedaliero-Universitaria S. Anna, 44124 Cona, Italy; giorgio.galoppini@edu.unife.it (G.G.); amarangoni021@gmail.com (A.M.); francescacirilli@hotmail.it (F.C.); francescaruffilli94@gmail.com (F.R.); carlo.garaffoni@unife.it (C.G.); gvl@unife.it (M.G.); ettore.silvagni@edu.unife.it (E.S.); 2 School of Medicine, University of Milano Bicocca, 20126 Milan, Italy; carlo.scire@unimib.it

**Keywords:** systemic lupus erythematous, multidisciplinary approach, multidisciplinary team, systematic literature review

## Abstract

Systemic lupus erythematosus (SLE) is characterized by multisystemic clinical manifestations ranging from a relatively mild involvement to potentially life-threatening complications. Due to this complexity, a multidisciplinary (MD) approach is the best strategy for optimizing patients’ care. The main aim of this systematic literature review (SLR) was to scrutinize the published data regarding the MD approach for the management of SLE patients. The secondary objective was to evaluate the outcomes of the MD approach in SLE patients. The Preferred Reporting Items for Systematic Reviews and Meta-Analysis guidelines were used. We performed an SLR to retrieve articles available in English or Italian listed in PubMed, Embase, Cinahl, and Cochrane Library concerning the MD approach used in observational studies and clinical trials. Four independent reviewers performed the study selection and data collection. Of 5451 abstracts evaluated, 19 studies were included in the SLR. The MD approach was most frequently described in the context of SLE pregnancy, reported in 10 papers. MD teams were composed of a rheumatologist, except for one cohort study; a gynecologist; a psychologist; a nurse; and other health professionals. MD approaches had a positive impact on pregnancy-related complications and disease flares and improved SLE psychological impact. Although international recommendations advise an MD approach for managing SLE, our review highlighted the paucity of data supporting this strategy, with most of the available evidence on the management of SLE during pregnancy.

## 1. Introduction

Systemic lupus erythematosus (SLE) is a chronic autoimmune disease typically characterized by unpredictable phases of remission and relapse. The disease can exhibit a fluctuating course, ranging from relatively mild involvement to potentially life-threatening complications. Owing to the therapeutic progress in the last few years, the survival rate of SLE patients has considerably increased; this has highlighted the importance of multifaceted care targeting not only the active disease but also its long-term complications, with particular attention to organ damage caused by the disease itself and by the chronic treatment [1].

Due to this complexity and the degree of potential multiorgan manifestations, a multidisciplinary (MD) approach involving other specialists in addition to the rheumatologist would theoretically be the best strategy for the optimal management of SLE. The MD team would vary according to the clinical phenotype of the disease, including different medical specialists and non-medical healthcare professionals.

Despite the a priori usefulness of a multi-professional approach to the diagnosis and treatment of SLE, no formalized clinical pathways and MD approaches to SLE are available. Based on the good evidence of the utility of the MD approach in oncology—where it has been proven to be effective in reducing time to diagnosis, improving staging process accuracy, and promoting tighter adherence to treatment guidelines [2]—we aimed to collect evidence on the MD approach in SLE through a systematic literature review (SLR) to inform MD team development for SLE care.

The primary aim of this SLR was to scrutinize the published data regarding the MD approach for the management of SLE patients. The secondary objective was to evaluate the outcomes of the MD approach in SLE patients in terms of disease activity, morbidity, patient’s reported outcomes, and measures of the costs of care.

## 2. Materials and Methods

The review was conducted according to the Preferred Reporting Items for Systematic Reviews and Meta-Analysis (PRISMA) statement [3]. The systematic review was registered in PROSPERO (ID = CRD42022309565).

Reports published up to 8th February 2022 were included; the search was performed in the Medline, Embase, Cinahl, and Cochrane Library databases, using the keywords “SLE”, “MD Team Meeting”, “MD Teams”, “Interdisciplinary Management”, and “Patient Outcomes” among others. Articles were screened according to the Population, Intervention, Comparison/Control, Outcome, and Study design (PICOS) framework: (P) population: patients with a clinical diagnosis of SLE (clinical diagnosis by an expert clinician or according to the American College of Rheumatology (ACR) 1997 criteria [4], American College of Rheumatology (ACR)/European League Against Rheumatisms (EULAR) 2019 criteria [5], or Systemic Lupus International Collaborating Clinics (SLICC) criteria [6]; (I) intervention: any MD management; (C) comparator: traditional approach/(other) multidisciplinary care/no comparator; (O) outcome (if available): SLE Disease Activity Index, morbidity, damage, mortality (overall survival), patient’s satisfaction, patient’s reported outcomes including health-related quality of life (HRQoL), quality indicators, and costs of care. Risk of bias was assessed according to the National Heart, Lung, and Blood Institute risk of bias tool for any type of study, and rated as high, low, or unclear [7]. The type of studies included were English or Italian systematic literature reviews, meta-analyses, RCTs, controlled trials, non-controlled trials, diagnostic accuracy studies, cohort studies, cross-sectional studies, case–control studies, and case series (>5 patients). The full search strategy is available in the online Supplemental Data S1.

Continuous variables are reported as mean (±SD) or as median (IQR) for non-normally distributed data. Categorical variables are reported as absolute and relative frequencies. Since a meta-analysis of data was not feasible, the overall effect of the MD approach in the different studies was summarized in a dichotomous way.

## 3. Results

**Figure 1:**
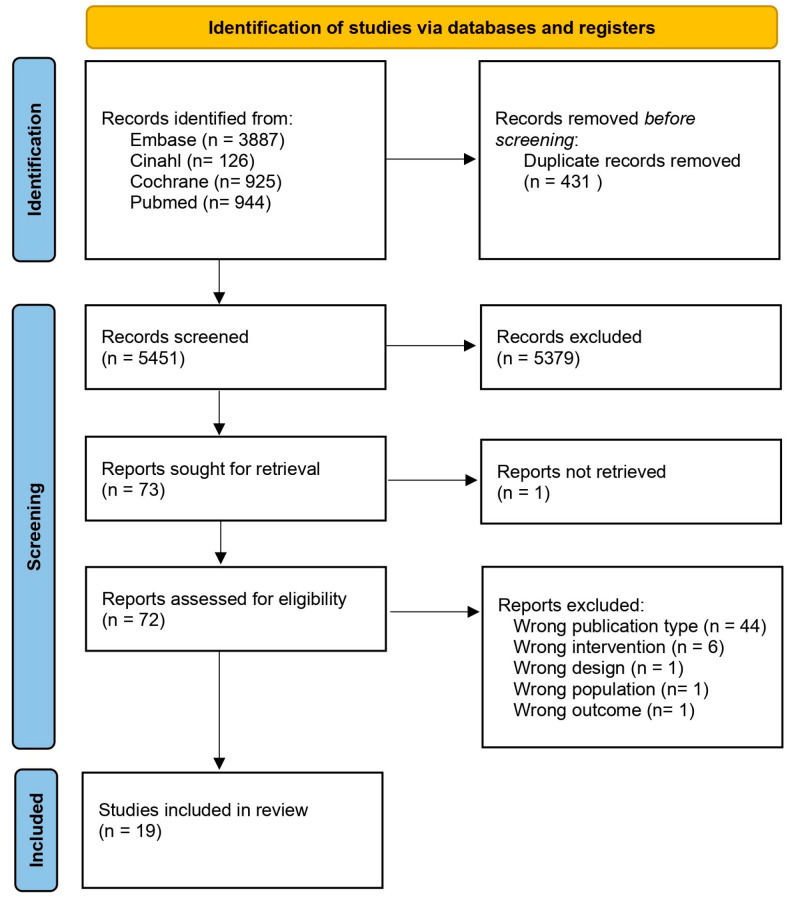
Literature retrieval strategy according to the Preferred Reporting Items for Systematic Reviews and Meta-Analysis (PRISMA) statement.

The search retrieved 944 citations from PubMed, 3887 from Embase, 925 from Cochrane, and 126 from Cinahl. After excluding duplicates, 5451 references were screened by title and abstract; 72 references (including 1 cross-reference) underwent full-text analysis. Data extraction from 19 papers was performed. Figure 1 summarizes the number of papers excluded and the reasons for exclusion. Analytical data are reported in Table 1.

### 3.1. Baseline Characteristics of Patients Included

In total, 1154 people were enrolled in studies comparing intervention and a control group (5 RCTs and 5 cohort studies); 498 patients (mean age: 36.18, SD ± 6.64 years) were included in intervention arms and 656 patients (mean age: 38.40 SD ± 6.77 years) in comparator arms. Two studies did not report the mean age of participants [13,16]. Median disease duration in intervention and comparator groups amounted to 9.5 years (IQR: 7.23–11.35) and 9 years (IQR: 7–10.25), respectively. Median follow-up time was 11.60 months (IQR: 9.55–12).

An additional 9 studies (8 cohort studies and 1 case series), without a comparator group, included 865 patients (median age: 32.90 SD ± 11.32 years), with a median disease duration of 6.33 years (IQR: 6.28–8.58). Median follow-up time was 11.63 months (IQR: 11.06–17.25). Caucasian ethnicity was the most frequently represented (included in 57.89% of all the reviewed studies); African American and Asian people were the second most represented groups (each present in 28.95% of the studies).

### 3.2. Risk of Bias

**Figure 2:**
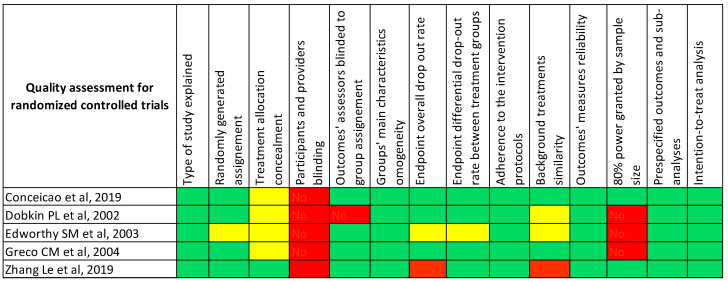

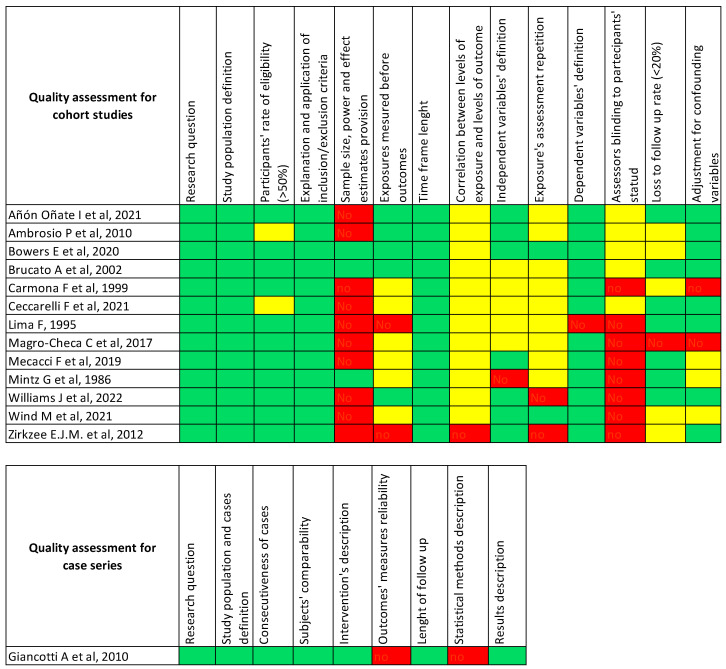
Quality assessment tables of included studies. Review of authors’ judgements about each risk of bias using NIH risk of bias tool. Green box = “yes/low risk of bias”; yellow box = “not applicable/not reported”; red box = “no/potential risk of bias” [8,9,10,11,12,13,14,15,16,17,18,19,20,21,22,23,24,25,26,27].

Altogether, the five RCTs showed a low risk of bias according to the National Heart, Lung, and Blood Institute risk of bias tool [7] (Figure 2). Of note, in none of the studies were the patients or the providers blinded to the assignment group, since interventions depended on the healthcare providers’ actions. A higher risk of bias in 11/13 cohort studies was determined due to the lack of sample size justification, power of study description, or variance and effect estimates.

### 3.3. Composition of the MD Team

The composition of the MD team varied among the included studies. A rheumatologist was the most frequently involved (18/19), followed by a gynecologist (10/19) and psychologist (4/19). Other professional figures were reported in 11 studies, including a hematologist (2/19), nephrologist (2/19), clinical nurse specialist (3/19), neurologist (2/19), and psychiatrist (2/19).

Pregnancy in lupus patients was the most frequent setting in which an MD approach was extensively applied. Ten studies described the management of pregnant lupus patients in a combined rheumatologic–obstetric clinic developed to provide correct pre-pregnancy counseling and prevent pregnancy complications. Other physicians attended this MD board, such as hematologists [19,24], nephrologists [21,24], and pediatricians [22]. Only the study by Wind et al. compared a prospective mixed cohort of 30 SLE/antiphospholipid syndrome (APS) patients (12 patients with SLE) managed in an MD clinical pathway with a retrospective mixed cohort of 48 SLE/APS patients (30 patients with SLE) managed according to usual care practices (historical pathway). The clinical pathway board included a rheumatologist and gynecologist, together with a nephrologist and thrombosis and hemostasis specialists; on-demand professionals included a pulmonologist, cardiologist, radiologist, and social worker, employed whenever deemed appropriate [24].

A total of 4 RCTs, involving 305 SLE patients, included a psychologist in the MD team [8,9,10,11]. Dobkin et al. designed an RCT in which 64 SLE patients underwent brief supportive–expressive group psychotherapy alongside standard care treatment and compared them with 69 SLE patients in the control arm [9]. A total of 32 SLE patients were enrolled in the intervention arm by Greco et al. in an RCT that aimed to evaluate the efficacy of biofeedback-assisted cognitive behavioral treatment in contrast to 27 SLE patients treated with usual care alone [11]. Conceição et al. evaluated the efficacy of psychoanalytic psychotherapy in 37 SLE patients in the intervention arm compared with 43 SLE patients in the control group receiving usual care alone [8].

Finally, one RCT involved a pharmacist in the MD group to increase patients’ knowledge of and adherence to prescribed drugs [12].

### 3.4. Characteristics of MD Intervention

The different MD teams mostly employed an approach that pivoted on the periodical standard clinical evaluation of the patients (11/19, complemented in 3/19 studies by psychotherapy sessions), regardless of the healthcare professionals involved. In 4/19 cases, intervention focused on gathering information through in-person meetings or phone calls, without the direct involvement of a rheumatologist [12,15,21,23]. Except for the management of pregnancy-related and psychological issues in SLE, we found heterogeneous examples of MD approaches to SLE patients. There was also great variability in terms of intervals between subsequent accesses by the MD groups. Patients were assessed at least monthly, ranging from weekly visits for group psychotherapy (3/19) to the variable intervals tailored according to the pregnancy week (8/19, the intervals becoming shorter as the delivery approached).

The management of pregnant SLE patients was similar throughout different studies. Follow-up visits were scheduled at established intervals; a rheumatologist, gynecologist, or both would evaluate the woman’s health. Periodical, instrumental examinations were also performed, such as ultrasonographic fetoplacental Doppler and fetal echocardiographic examinations (i.e., cardiotocography) [14,17,19,21,22,26].

Interventions aiming at improving the management of psychological aspects of SLE comprised both group (2/4) [9,10] and individualized (2/4) [11,27] psychoeducational/psychological therapy, and even sessions of cognitive behavioral training.

One study described the effects of a pre-clinical intervention. First, the data from insurance policies allowed the identification of SLE patients at higher risk of inappropriate use of healthcare services (such as emergency departments) through a mixed machine-learning-based algorithm and the collection of patients’ primary care physicians’ opinions. Patients were then referred to a nurse leading the integrated care management program who evaluated each patient’s health status and coordinated his/her care needs. The rheumatologist did not evaluate the patients systematically unless the leading nurse deemed it necessary; otherwise, the patient was addressed to the most appropriate figure, including social workers, community resource specialists, or pharmacists [23].

Laboratory tests were performed in more than half of the studies (11/19), the majority including complete blood count and liver and/or kidney function tests (7/11); in pregnancy studies, biomarkers of SLE disease activity were dosed at least once during the pregnancy period. The timing of the re-test ranged from one to three months, with two studies scheduling at least one laboratory assessment [19,26]. Instrumental investigations were also performed (10/19), nearly all in the setting of SLE pregnancies; 7/19 were non-invasive, ultrasound-based techniques. Finally, in 2/19 a 3-tesla MRI of the brain was performed at baseline [25], in 1 case even after 3–18 months, enabling the same MD team to evaluate the evolution of previous neurological manifestations [20].

### 3.5. Outcomes Evaluated

Different studies evaluated the efficacy of the MD approach through different outcomes. The assessment of SLE disease activity was the most frequently reported outcome (13/19), expressed as clinically defined new flares (7/10) [14,17,19,21,22,24,26] and through clinimetric indexes (6/10) [9,10,11,12,18,27].

Due to the high number of studies in SLE pregnancies, maternal–fetal outcomes (i.e., thromboembolic manifestations, pregnancy, delivery, or post-partum complications were represented in the majority of cases (10/19). Measures of cumulative damage were reported in two studies, in both cases with the SLICC damage index (SDI); no statistically significant intragroup or intergroup changes emerged [8,12,28].

**Figure 3:**
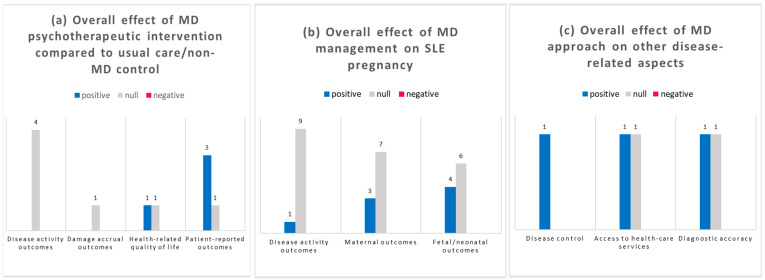
Qualitative representation of the overall effect of the MD approach on SLE outcomes in the context of psychiatry (**a**), pregnancy (**b**), and other dimensions of the disease (**c**). Where the authors did not make a comparison with a control group, the effect of the interventions was considered null. (**a**) Effects of different psychological/psychiatric interventions on SLE disease activity, damage accrual, health-related quality of life, and patient-reported outcomes, compared with patients undergoing usual care. (**b**) In the context of SLE pregnancy, the comparison group consisted of other pregnant SLE patients (treated according to site-specific usual care procedures) or non-SLE pregnant women. If a comparator group was absent or the effect of MD was not significantly different from that of a usual care approach, the effect was considered null. (**c**) Three studies evaluated multidisciplinary experiences in the context of disease control and compliance to therapy [12], access to healthcare services [15,23], or attribution of a neuropsychiatric event to SLE [20].

Health-related quality of life (4/19) [9,11,12,27], patient satisfaction (2/19) [9,11,12,27], and patient-reported outcomes (5/19) [9,10,11,12,27] were determined through different tools and therefore were not directly comparable. No study examined the impact of the MD approach on the direct and indirect costs of the disease. The overall effect of the MD approach in the different studies is summarized in Figure 3.

#### 3.5.1. Pregnancy Outcomes

In a prospective study conducted from 1974 to 1983 [22], miscarriage occurred in 16% of SLE pregnancies compared with 5.7% of healthy controls (*p* < 0.009). Subgroup analyses found that premature births were higher in mothers with active SLE (at conception or during pregnancy) than with inactive SLE (59% vs. 39%, respectively, *p* < 0.0001). Cases of small newborns for gestational age (SGA) occurred more frequently in SLE women than in controls (23% vs. 4.2%, *p* < 0.0001) but disproportionately between active and inactive SLE (65% vs. 35%, respectively). Unlike the other studies illustrated below, in this cohort the MD approach was not meant to control disease activity but to reduce stillbirth through cesarean section when fetal distress occurred. In a study conducted by Ceccarelli et al., none of the outcomes significantly differed between SLE pregnancies (managed by an MD team involving rheumatologists and gynecologists) and healthy women’s pregnancies, except for SGA/lower weight at birth (which were more frequent in the SLE cohort), including miscarriage (11.4% vs. 12%, *p* = n.s.), preterm birth (25.7% vs. 19%), gestational hypertension (7.1% vs. 3%), preeclampsia (2.9% vs. 1%), and Intrauterine Growth Restriction (IUGR, 5% vs. 1%) [18]. The study by Añón et al. compared the effects of traditional versus MD management of pregnancies in patients affected by various rheumatic diseases, finding that total miscarriages accounted for 56.3% vs. 3% (*p* < 0.001), respectively. Of note, SLE and APS patients represented the groups with higher percentages of miscarriage, at 40.8% and 87.5%, respectively. The frequency of adverse neonatal outcomes in standard care was 42.8% in SLE and 90% in APS. However, after the introduction of the MD team, the risk of adverse neonatal outcomes reduced to 36.7% (95% CI: 21.3–52.1) in the SLE group and 87.5% (95% CI: 77–98) in the APS group [13]. Similar results were observed by Wind et al., who found that maternal outcomes in SLE and primary/secondary APS patients were 36/71 in the historically treated cohort versus 22/41 in the MD-treated one (OR 0.91, 95% CI 0.38–2.17). This study also reported the incidence of SLE flares during pregnancy, being less frequent in the MD cohort (12.5% vs. 39.5%, OR 0.22, 95% CI 0.04–1.09) [24]. SLE exacerbations were also recorded in Mintz’s, Giancotti’s, and Ceccarelli’s studies at 59.7%, 20%, and 40%, respectively [18,22,26].

#### 3.5.2. Disease Activity

Four studies monitored variations in disease activity in the setting of MD psychoanalytic therapy applied to SLE management, using the SLE Disease Activity Index (SLEDAI) (3/4), SLAM-R (3/4), or both (2/4). A mean intragroup reduction of 1.77 SLAM-R points from baseline to 9-month assessment was observed by Greco et al. for the intervention arm, with statistical intragroup significance [11]. Nevertheless, the MD team did not demonstrate a significant effect on disease activity expressed as SLAM-R [9,11] or SLEDAI [8] between the intervention and control groups. By contrast, in the study by Zhang et al., exploring the usefulness of the inclusion of a pharmacist in the MD team, patients in the intervention arm showed a significantly lower SLEDAI score at 12 months follow-up when compared with the control group (0 vs. 2, *p* = 0.027) [12]. Two studies reported the impact of an MD team on SDI for intervention and control groups, both at baseline and at the end of follow-up, without any evidence of an additional benefit from the MD approach [8,12].

#### 3.5.3. Patient-Reported Outcomes

Four studies evaluated HRQoL as a primary outcome; all of them were RCTs, and in 3/4 the MD team involved a psychologist. Dobikn et al. used the Short-Form 36 Health Survey (SF-36) [29], a 36-item score that measures 8 aspects of health and wellbeing [9], in an RCT involving 133 SLE patients, with the aim to evaluate the therapeutic effect of brief supportive–expressive therapy. No significant difference was observed between the intervention and comparator groups in SF-36 scores at the end of follow-up: treatment versus control SF-36 Physical Component Summary (PCS) score difference: 0.6 (95% CI −2.8, 4); SF-36 Mental Component Summary (MCS) score difference: −2.4 (95% CI −6.4, 1.6). Greco et al. used the physical function scale (PFS) of SF-36 as a primary outcome in an RCT evaluating the efficacy of biofeedback-assisted cognitive behavioral treatment programs for SLE patients. In total, 32 patients in the intervention arm had significantly greater improvement than the control group in terms of SF-36 PCS score at 9 months of follow-up (*p* = 0.023); in the control group, the SF-36 score at the end of follow-up was significantly better (*p* = 0.036) than baseline as well [11].

Systemic Lupus Erythematosus Quality of Life (SLEQOL), a self-reported questionnaire evaluating the SLE quality of life [30] was used by Conceição et al. to evaluate the effect of psychoanalytic psychotherapy on SLE patients. The SLE patients were randomized into an intervention arm (37 patients), in which they were treated with a short-term therapy (derived from psychoanalysis) alongside usual care, and a control group (43 patients), which was treated with usual care alone. The intervention arm showed significant improvement in SLEQOL score at the end of the follow-up compared with the baseline (*p* < 0.001) and a significant difference compared with the control group (*p* = 0.043) [8].

Zhang et al. evaluated the impact of an MD team on HRQoL in SLE patients using EQ5DL to measure health status; patients in the intervention arm improved in the EQ5D index, which increased from a median of 0.81 at baseline to a median of 0.94 at the end of 12 months of follow-up (*p* = 0.006) [12].

Five studies evaluated the effect of MD care on patient-reported outcomes (PROs): all of them were RCTs, four of which focused on psychological support. Two studies evaluated PROs focusing on depressive symptoms in SLE patients managed with psychological support alongside usual care: Greco et al. found a significant difference between the intervention (32 patients) and control group (27 patients) in depressive symptoms measured with the Center for Epidemiological Studies Depression Scale (CESD), favoring the intervention arm (*p* = 0.012); Conceição et al. used the Hospital Anxiety and Depression Score (HADS) in their RCT and found a significantly better score in the intervention arm (37 patients) than the control group (43 patients) (*p* = 0.019).

Pain was evaluated by Greco et al. using the revised Arthritis Impact Measurement Scales, pain subscale (AIMS2-Pain) and Multidimensional Pain Inventory (MPI); the MD approach involving psychological support showed a significant reduction in AIMS-2 PAIN and MPI scores in the intervention group immediately after treatment (*p* = 0.028), although this effect was not maintained at 9 months follow-up (*p* = 0.305). Zhang et al. used PROs to evaluate patients’ compliance using compliance questionnaire rheumatology (CQR): MD care showed no significant improvement in CQR score when compared with the control group.

#### 3.5.4. Other Outcomes

The diagnostic utility of an MD board in the context of neuropsychiatric SLE (NPSLE) was investigated for the first time in a cohort of SLE patients (prospectively followed in the NPSLE Clinic of the Leiden University Medical Centre between 2007 and 2009) by Zirkzee and colleagues [25]. They described MD consensus as a standard for diagnosing and defining phenotypes in NPSLE. Magro-Checa et al. continued analyses in the same cohort of SLE patients until 2016, evaluating the reliability of an MD approach in the SLE attribution process of neuropsychiatric involvement. First, they calculated the concordance between the first visit assessment and the end of follow-up in the attribution of a neuropsychiatric event to SLE, finding an agreement (expressed as Cohen’s kappa coefficient) of 0.82. Then, they compared the performance of this strategy with other attribution models; a higher concordance emerged between the MD strategy and the Italian NPSLE algorithm, with a coefficient of 0.59 (reassessment versus algorithm) [20].

## 4. Discussion

SLE is an autoimmune condition that necessitates comprehensive management due to its multisystemic and multidimensional involvement, as underscored by European recommendations [31,32]. While many hospitals have rheumatologists and dedicated settings, such as Lupus clinics, committed to managing SLE patients with on-demand support from other specialists, standardized MD teams are not always available. The limited number of studies in this field retrieved by our SLR may be attributed to the underreporting of existing MD teams in the scientific literature, as well as the lack of such MD teams, resulting in a shortage of rigorous research on this topic.

Although it is well known that many organs and systems can be affected in SLE, we observed an imbalance in data focused on SLE pregnancy and (to a lesser extent) psychological aspects; surprisingly, none of the described MD teams analyzed included, for instance, a cardiologist, pulmonologist, or dermatologist, and a nephrologist and hematologist were cited only twice each [13,19,24]. Moreover, in only 22% of reviewed studies did the MD teams include more than 1 medical professional (other than a rheumatologist) at the same time. That was the case for Magro-Checa et al., who demonstrated that the concomitant inclusion of specialists in neurology, psychiatry, and vascular medicine in the diagnostic process of suspected NPSLE is crucial for a more accurate attribution of neuropsychiatric symptoms to SLE. This was proved more clearly when the cases were reassessed by the same MD group after an interval of at least 3 months [20]. Early recognition of organ involvement is a mainstay in treating many autoimmune diseases, aiming at the prevention of complications. However, we could not ascertain if the MD approach provided better results (in terms of disease activity or damage accrual) when applied at the beginning rather than years after SLE diagnosis, due to great variability in disease duration among patients included in the studies. Tight intervention is crucial in SLE pregnancies too, and Wind’s study clearly demonstrated that MD management of pregnant SLE patients effectively reduced the number of SLE flares (and, indirectly, adverse fetal outcomes) [24].

In our SLR, the included studies provided mainly qualitative assessments; in this respect, only RCTs expressed disease activity as a clinimetric measure (through SLEDAI or SLAM-R tools), whereas cohort studies assessed only the occurrence and the number of flares, with rare exceptions. Outcome measures were highly heterogeneous between studies, many of which focused on SLE patients’ pregnancies. Therefore, pregnancy, delivery, and newborn outcomes acquired the greatest importance, confirming the improvements achieved in this field over the years, also thanks to the MD approach. In these studies, SLE disease activity was mentioned only in cases of new flares and mainly assessed clinically, without clinimetric measures explicated. The study by Ceccarelli et al. represented an exception here since they reported SLEDAI-2K measurements at the beginning and before the end of pregnancy, but without finding any difference [18].

By contrast, clinimetric disease activity measurements were thoroughly reported in studies evaluating psychological procedures, although they did not provide any significant effect. Besides aiming for low disease activity, an MD approach could achieve a better quality of life in SLE patients. Hence, as expected, interventions focusing on improving the ability to cope with disease burden were more effective on quality-of-life-related scores and PROs than disease activity indexes.

According to the above-stated assumption, it is surprising that the inclusion of a pharmacist in an MD board could provide a reduction in SLEDAI in the intervention group in the trial conducted by Zhang et al. [12]. This is convincingly explained by the authors through evidence of better adherence to pharmacological treatment among patients who were educated by pharmacists. Compliance with therapy was assessed both directly, through self-reported questionnaires, and indirectly, through patient-reported questionnaires inquiring about satisfaction with therapy and relative information obtained; although no statistical intergroup difference emerged in the former, a strongly significant difference in favor of the treatment group was highlighted in the latter case.

In terms of damage accrual outcomes (expressed as SLICC-SDI), neither in Zhang’s nor in Conceição’s study was a significant variation observed; nonetheless, given that SDI is a measure of damage accrued over a long time, its variations are hardly seen during the span of trials or observational studies [12,27].

Our SLR has some limitations. We observed significant heterogeneity in terms of study design, control arms (sometimes consisting of healthy subjects, in other cases of SLE patients), disease activity indices, and outcome measures. Therefore, a meta-analysis of outcomes from the different studies was not possible, even when the same outcome measures were recorded.

Nonetheless, our SLR unequivocally highlights how useful an MD approach can be for the optimal management of a complex disease such as SLE. However, there are still many unmet needs. Too few studies involved nephrologists and hematologists, which is surprising considering how frequently SLE affects the kidneys and blood cells. Moreover, there were no retrieved reports describing MD teams with a cardiologist or an infectious disease specialist; this was once again unexpected, considering the pivotal role of cardiovascular and infectious risk management in this condition. These observations may be partly attributed to a prevalent organ-based care approach in SLE, wherein patients are managed exclusively by specialists focused on the affected organ (e.g., relying on a dermatologist for cutaneous SLE or a nephrologist for renal involvement). The optimal implementation of an MD approach should consistently involve the inclusion of a rheumatologist, thus promoting comprehensive and holistic patient-centered management. The current recommendations do not mention a rheumatologist as the referring physician for the care of SLE patients, despite emphasizing the need for MD collaboration among specialists in shared patient–physician decision making. However, it is crucial to recognize that a rheumatologist should always play a pivotal role within an MD team dedicated to SLE treatment, given their extensive knowledge of the protean manifestations of the disease.

In our opinion, an effective MD approach should distinguish between the management of the acute and chronic phases of SLE. During the acute phase, appropriate specialists should be involved based on the specific disease phenotype and manifestations (e.g., a nephrologist for lupus nephritis). In the chronic phase, other healthcare professionals (e.g., specialized nurses and psychologists) can improve quality of life by managing aspects related to disease chronicity. In the current era of improved life expectancy for SLE patients, preventing comorbidities and damage is crucial and underscores the importance of the MD approach. Therefore, many efforts should be made to promote the establishment of MD teams and which include those professionals who have remained to date on the margins of SLE patients’ collaborative management. Efforts have been made by the European Commission through the creation of European Reference Networks (ERNs)—one of which pertains to rare connective tissue diseases (including SLE)—aiming at creating organizational reference models of care processes (called patient care pathways, PCPs) that can be shared by connecting healthcare providers across Europe. Moreover, PCPs should link different specialists through member centers in order to facilitate knowledge dissemination for rare connective tissue diseases such as SLE [33].

## 5. Conclusions

In conclusion, promoting official MD teams is advisable for successful collaboration among specialists and better healthcare for SLE patients. Synergistic interactions among different specialists in the MD team, under the direction of the rheumatologist, may enhance professional development, decision making consistency, and shared responsibility.

## Figures and Tables

**Table 1:** Characteristics and results of selected studies. Randomized controlled trial (RCT); retrospective cohort study (RC); prospective cohort study (PC); case series. Systemic Lupus Erythematosus (SLE) Disease Activity Index (SLEDAI); Systemic Lupus International Collaborating Clinics (SLICC) Damage Index (DI); American College of Rheumatology (ACR); Systemic Lupus Activity Measure (SLAM); SLE Quality Of Life (SLEQOL); Short-Form 36 Health Survey (SF-36); Intrauterine Growth Restriction (IUGR); neuropsychiatric SLE (NPSLE); revised Arthritis Impact Measurement Scales, pain subscale (AIMS2-Pain); Center for Epidemiological Studies Depression Scale (CES-D); multidisciplinary (MD).

First Author, Year, Type of Study	Number of Participants, Intervention |Control	Mean Age (Years), Intervention |Control	Mean Disease Duration (Months), Intervention |Control	Physicians Involved in the MD Team (Non-Medical Professionals)	Type of Intervention	Outcome
Conceicao, 2019, RCT [8]	80, 37|43	42|42.7	148|139	Rheumatologist, Psychologist	Group psychotherapy	SLEDAI; SLICC-ACR-DI; SLEQOL
Dobkin PL, 2002, RCT [9]	133, 64|69	42|43	136|126	Rheumatologist, Psychologist	Group psychotherapy	Disease activity, health quality, social and psychological support
Edworthy, 2003, RCT [10]	124, 58|66	42.5, 42|43	130, 137|126	Rheumatologist, Psychologist	Group psychotherapy	SLAM; illness intrusiveness
Greco, 2004, RCT [11]	92, 32|60	48.2|47	120|96	Rheumatologist, Psychologist	Biofeedback cognitive behavioral treatment	SLAM; SF-36; pain (AIMS2-Pain); depression (CES-D)
Zhang LE, 2019, RCT [12]	82, 42|40	31.5|30.3	-	Rheumatologist, (Pharmacist, Nurse)	Pharmacist-led multidisciplinary care	SLEDAI, SLICC-SDI, quality of life, and patient-reported outcomes
Anon, 2021, RC [13]	49	-	-	Rheumatologist, Gynecologist	Clinical, laboratory, and instrumental pregnancy follow-up by a rheumatological–gynecological team	Materno-fetal outcomes
Ambrosio, 2010, RC [14]	107	29	75.6	Rheumatologist, Obstetricians	Clinical, laboratory, and instrumental pregnancy follow-up by a rheumatological–gynecological team	Pregnancy complications, delivery outcome, newborn outcome
Bowers E, 2019, PC [15]	104, 56|48	39.4|43.4	-	Rheumatologist, Faculty Physician (Nurse)	Phone call by a nurse 48 h after hospital discharge	Hospital readmission rate
Brucato A, 2002, RC-PC [16]	111	-	-	Rheumatologist, Gynecologist	Clinical, laboratory, and instrumental pregnancy follow-up by a rheumatological–gynecological team	Obstetric and fetal outcomes, flares during pregnancy
Carmona F, 1999, PC [17]	46	28.6	75	Rheumatologist, Obstetricians	MD approach during pregnancy	Obstetric outcomes, flares during pregnancy
Ceccarelli F, 2021, PC [18]	150, 50|100	33|31	72	Rheumatologist, Gynecologist	Pre-gestational counseling and a multidisciplinary approach during pregnancy	Obstetric and fetal outcomes, disease activity
Lima, 1995, PC [19]	90	30.7	76	Rheumatologist, Gynecologist, Hematologist	Clinical, laboratory, and instrumental pregnancy follow-up by a rheumatological–gynecological team	Fetal outcomes: successful pregnancies, prematurity, IUGR. Maternal outcomes: SLE flares
Magro-Checa, 2017, PC [20]	304	42.5	55.2	Rheumatologist, Neurologist, Psychiatrist, Vascular Physician	MD assessment in suspected NPSLE	Correct attributions of NP events
Mecacci F, 2019, RC [21]	86, 27|59	32.1|34.1	99.6|68.4	Rheumatologist, Gynecologist, Nephrologist	Clinical, laboratory, and instrumental pregnancy follow-up by a rheumatological–gynecological team	Disease flare (especially renal), maternal–fetal outcomes (especially preeclampsia and IUGR)
Mintz G, 1986, PC [22]	225, 102|123	27.2|30.3	58.8|-	Rheumatologist, Gynecologist, Neonatologist	Clinical, laboratory, and instrumental pregnancy follow-up by a rheumatological–gynecological team	Disease activity (clinically attested), maternal–fetal outcomes
Williams J, 2022, PC [23]	67	60	180	Rheumatologist (only if needed), (Nurse, Pharmacist, Community Resource Specialist)	Referral of the patient to the most appropriate professional	Risk of potentially avoidable healthcare service use
Wind M, 2021, RC-PC [24]	78, 30|48	32|31	108|108	Rheumatologist, Gynecologist, Nephrologist, Hematologist	Clinical, laboratory, and instrumental pregnancy follow-up by a rheumatological–gynecological team	Disease flares and maternal–fetal outcomes
Zerkzee EJM, 2012, PC [25]	71	42	101	Rheumatologist, Neuropsychologist, Neurologist, Psychiatric, Radiologist, and Resident in Internal Medicine	MD assessment in suspected NPSLE	Diagnosis of neuropsychiatric SLE and classification into 3 different phenotypes (ischemic, inflammatory, and undefined)
Giancotti, 2010, CS [26]	20	32.9	104.16	Rheumatologist, Gynecologist	Clinical, laboratory, and instrumental pregnancy follow-up by a rheumatological–gynecological team	Maternal outcomes: number of SLE flares

## Data Availability

No new data were created or analyzed in this study. Data sharing is not applicable to this article.

## Supplementary Materials

The following supporting information can be downloaded at: https://www.mdpi.com/article/10.3390/jcm12124059/s1. Supplemental Data S1: The full search strategy [34,35].

## References

[1] Kiriakidou M., Ching C.L. (2020). Systemic Lupus Erythematosus. Ann. Intern. Med..

[2] Pillay B., Wootten A.C., Crowe H., Corcoran N., Tran B., Bowden P., Crowe J., Costello A.J. (2016). The impact of multidisciplinary team meetings on patient assessment, management and outcomes in oncology settings: A systematic review of the literature. Cancer Treat. Rev..

[3] Page M.J., McKenzie J.E., Bossuyt P.M., Boutron I., Hoffmann T.C., Mulrow C.D., Shamseer L., Tetzlaff J.M., Akl E.A., Brennan S.E. (2021). The PRISMA 2020 statement: An updated guideline for reporting systematic reviews. PLOS Med..

[4] Low E.S.H., Krishnaswamy G., Thumboo J. (2019). Comparing the 1997 update of the 1982 American College of Rheumatology (ACR-97) and the 2012 Systemic Lupus International Collaborating Clinics (SLICC-12) criteria for systemic lupus erythematosus (SLE) classification: Which enables earlier classification of SLE in an urban Asian population?. Lupus.

[5] Aringer M., Costenbader K., Daikh D., Brinks R., Mosca M., Ramsey-Goldman R., Smolen J.S., Wofsy D., Boumpas D.T., Kamen D.L. (2019). 2019 European League Against Rheumatism/American College of Rheumatology classification criteria for systemic lupus erythematosus. Ann. Rheum. Dis..

[6] Petri M., Orbai A.-M., Alarcón G.S., Gordon C., Merrill J.T., Fortin P.R., Bruce I.N., Isenberg D., Wallace D.J., Nived O. (2012). Derivation and validation of the Systemic Lupus International Collaborating Clinics classification criteria for systemic lupus erythematosus. Arthritis Rheum..

[7] Study Quality Assessment Tools. NHLBI, NIH. https://www.nhlbi.nih.gov/health-topics/study-quality-assessment-tools.

[8] Conceição C.T.M., Meinão I.M., Bombana J.A., Sato E.I. (2019). Psychoanalytic psychotherapy improves quality of life, depression, anxiety and coping in patients with systemic lupus erythematosus: A controlled randomized clinical trial. Hortic. Bras..

[9] Dobkin P.L., Da Costa D., Joseph L., Fortin P.R., Edworthy S., Barr S., Ensworth S., Esdaile J.M., Beaulieu A., Zummer M. (2002). Counterbalancing patient demands with evidence: Results from a pan-canadian randomized clinical trial of brief supportive-expressive group psychotherapy for women with systemic lupus erythematosus. Ann. Behav. Med..

[10] Edworthy S.M., Dobkin P.L., Clarke A.E., Da Costa D., Dritsa M., Fortin P.R., Barr S., Ensworth S., Esdaile J.M., Beaulieu A. (2003). Group psychotherapy reduces illness intrusiveness in systemic lupus erythematosus. J. Rheumatol..

[11] Greco C.M., Rudy T.E., Manzi S. (2004). Effects of a stress-reduction program on psychological function, pain, and physical function of systemic lupus erythematosus patients: A randomized controlled trial. Arthritis Rheum..

[12] Zhang L., Geng S., Qian L., Ye S., Wang X., Lu G., Ding Y., Li T. (2019). Multidisciplinary care in patients with systemic lupus erythematosus: A randomized controlled trial in China. Int. J. Clin. Pharm..

[13] Añón-Oñate I., Cáliz-Cáliz R., Rosa-Garrido C., Pérez-Galán M.J., Quirosa-Flores S., Pancorbo-Hidalgo P.L. (2021). Multidisciplinary Unit Improves Pregnancy Outcomes in Women with Rheumatic Diseases and Hereditary Thrombophilias: An Observational Study. J. Clin. Med..

[14] Ambrósio P., Lermann R., Cordeiro A., Borges A., Nogueira I., Serrano F. (2010). Lupus and Pregnancy—15 Years of Experience in a Tertiary Center. Clin. Rev. Allergy Immunol..

[15] Bowers E., Griffith M., Weinstein E., Pearson D., Kolfenbach J. (2019). A quality improvement intervention to reduce 30-day hospital readmission rates among patients with systemic lupus erythematosus. Arthritis Rheumatol..

[16] Brucato A., Doria A., Frassi M., Castellino G., Franceschini F., Faden D., Pisoni M.P., Solerte L., Muscarà M., Lojacono A. (2002). Pregnancy outcome in 100 women with autoimmune diseases and anti-Ro/SSA antibodies: A prospective controlled study. Lupus.

[17] Carmona F., Font J., Cervera R., Muñoz F., Cararach V., Balasch J. (1999). Obstetrical outcome of pregnancy in patients with systemic Lupus Erythematosus. A study of 60 cases. Eur. J. Obstet. Gynecol. Reprod. Biol..

[18] Ceccarelli F., Pirone C., Perricone C., Selntigia A., Orefice V., Pacucci V.A., Truglia S., Spinelli F.R., Galoppi P., Alessandri C. (2021). Pregnancy outcome in systemic lupus erythematosus patients: A monocentric cohort analysis. Rheumatology.

[19] Lima F., Buchanan N.M., Khamashta M.A., Kerslake S., Hughes G.R. (1995). Obstetric outcome in systemic lupus erythematosus. Semin. Arthritis Rheum..

[20] Magro-Checa C., Zirkzee E.J., De Voorde L.J.B.-V., Middelkoop H.A., Van Der Wee N.J., Huisman M.V., Eikenboom J., Kruyt N.D., Van Buchem M.A., Huizinga T.W. (2017). Value of multidisciplinary reassessment in attribution of neuropsychiatric events to systemic lupus erythematosus: Prospective data from the Leiden NPSLE cohort. Rheumatology.

[21] Mecacci F., Simeone S., Cirami C.L., Cozzolino M., Serena C., Rambaldi M.P., Gallo P., Emmi L., Cammelli D., Mello G. (2019). Preeclampsia in pregnancies complicated by systemic lupus erythematosus (SLE) nephritis: Prophylactic treatment with multidisciplinary approach are important keys to prevent adverse obstetric outcomes. J. Matern. Fetal Neonatal Med..

[22] Mintz G., Niz J., Gutierrez G., Garcia-Alonso A., Karchmer S. (1986). Prospective study of pregnancy in systemic lupus erythematosus. Results of a multidisciplinary approach. J. Rheumatol..

[23] Williams J.N., Taber K., Huang W., Collins J., Cunningham R., McLaughlin K., Vogeli C., Wichmann L., Feldman C.H. (2022). The Impact of an Integrated Care Management Program on Acute Care Use and Outpatient Appointment Attendance Among High-Risk Patients With Lupus. ACR Open Rheumatol..

[24] Wind M., Hendriks M., van Brussel B.T.J., Eikenboom J., Allaart C.F., Lamb H.J., Siebelink H.-M.J., Ninaber M.K., van Geloven N., van Lith J.M.M. (2021). Effectiveness of a multidisciplinary clinical pathway for women with systemic lupus erythematosus and/or antiphospholipid syndrome. Lupus Sci. Med..

[25] Zirkzee E.J., Steup-Beekman G.M., Van Der Mast R.C., Bollen E.L., Van Der Wee N.J.A., Baptist E., Slee T.M., Huisman M.V., Middelkoop H.A., Luyendijk J. (2012). Prospective Study of Clinical Phenotypes in Neuropsychiatric Systemic Lupus Erythematosus; Multidisciplinary Approach to Diagnosis and Therapy. J. Rheumatol..

[26] Giancotti A., Spagnuolo A., D’Ambrosio V., Pasquali G., Muto B., De Gado F. (2010). Pregnancy in lupus patients: Our experience. Minerva Obstet. Gynecol..

[27] Conceicao C., Meinao I., Blay S., Sato E. (2018). Brief group psychoanalytic psychotherapy improves qualityof life in patientswith systemic lupus erythematosus: A long-term follow-up study. J. Clin. Rheumatol..

[28] Romero-Diaz J., Isenberg D., Ramsey-Goldman R. (2011). Measures of adult systemic lupus erythematosus: Updated version of British Isles Lupus Assessment Group (BILAG 2004), European Consensus Lupus Activity Measurements (ECLAM), Systemic Lupus Activity Measure, Revised (SLAM-R), Systemic Lupus Activity Questionnaire for Population Studies (SLAQ), Systemic Lupus Erythematosus Disease Activity Index 2000 (SLEDAI-2K), and Systemic Lupus International Collaborating Clinics/American College of Rheumatology Damage Index (SDI). Arthritis Care Res..

[29] Ware J.E., Sherbourne C.D. (1992). The MOS 36-item short-form health survey (SF-36). I. Conceptual framework and item selection. Med. Care.

[30] Leong K.P., Kong K.O., Thong B., Koh E.T., Lian T.Y., Teh C.L., Cheng Y.K., Chng H.H., Badsha H., Law W.G. (2005). Development and preliminary validation of a systemic lupus erythematosus-specific quality-of-life instrument (SLEQOL). Rheumatology.

[31] Fanouriakis A., Kostopoulou M., Cheema K., Anders H.-J., Aringer M., Bajema I., Boletis J., Frangou E., Houssiau F.A., Hollis J. (2020). 2019 Update of the Joint European League Against Rheumatism and European Renal Association–European Dialysis and Transplant Association (EULAR/ERA–EDTA) recommendations for the management of lupus nephritis. Ann. Rheum. Dis..

[32] Fanouriakis A., Kostopoulou M., Alunno A., Aringer M., Bajema I., Boletis J.N., Cervera R., Doria A., Gordon C., Govoni M. (2019). 2019 update of the EULAR recommendations for the management of systemic lupus erythematosus. Ann. Rheum. Dis..

[33] Talarico R., Cannizzo S., Lorenzoni V., Marinello D., Palla I., Pirri S., Ticciati S., Trieste L., Triulzi I., Terol E. (2020). RarERN Path: A methodology towards the optimisation of patients’ care pathways in rare and complex diseases developed within the European Reference Networks. Orphanet J. Rare Dis..

[34] Harris P.A., Taylor R., Thielke R., Payne J., Gonzalez N., Conde J.G. (2009). Research electronic data capture (REDCap)—A metadata-driven methodology and workflow process for providing translational research informatics support. J. Biomed. Inform..

[35] Harris P.A., Taylor R., Minor B.L., Elliott V., Fernandez M., O’Neal L., Mcleod L., Delacqua G., Delacqua F., Kirby J. (2019). The REDCap consortium: Building an international community of software platform partners. J. Biomed. Inform..

