# Supplementary material for: Optimizing Patient Care: A Systematic Review of Multidisciplinary Approaches for SLE Management
Source: J Clin Med. 2023 Jun 15;12(12):4059. doi: 10.3390/jcm12124059 (PMC10298977; doi:10.3390/jcm12124059)
Supplement: Supplementary file 1 [file jcm-12-04059-s001.zip › jcm-2414275-supplementary.pdf]

Journal of
Clinical Medicine

MDPI

Supplementary material

Optimizing patient care: a systematic review of multidisciplinary approaches for SLE management

Giorgio Galoppini[1], Antonio Marangoni[1], Francesca Cirilli[1], Francesca Ruffilli[1], Carlo Garaffoni[1], Marcello Govoni[1], Carlo Alberto Scirè[2], Ettore Silvagni[1], Alessandra Bortoluzzi[1*]

[1] Section of Rheumatology, Department of Medical Sciences, University of Ferrara and Azienda Ospedaliera-Universitaria di Ferrara, Cona (Fe), Italy
[2] School of Medicine, University of Milano Bicocca, Milan, Italy.
* Correspondence: Alessandra Bortoluzzi, Rheumatology Unit, Department of Medical Sciences, University of Ferrara, Ferrara, Italy. Email: brtlsn1@unife.it

SUPPLEMENTAL MATERIAL

The review was conducted according to the Preferred Reporting Items for Systematic reviews and Meta-Analysis (PRISMA) statement. The systematic review was registered in PROSPERO (ID=CRD42022309565). Patients were not asked to firm an informed consent form, and the protocol did not undergo Ethical Committee approval, since this was a systematic literature review.

The search strategy was articulated into three steps.

At first, a systematic literature review was performed using electronic databases Medline, Embase, Cinhal and Cochrane Library. The search was completed on 8th February 2022. The search strategy was elaborated to include the greatest number of references dealing with the populations and the interventions object of the study by using the following keywords in combination with the Boolean operators OR and AND: “Lupus Erythematosus, Systemic”, “Multidisciplinary Team Meeting”, “MD Teams”, “Interdisciplinary Management”, “Patient Outcomes”, “Patient Management”, “Patient assessment” and “Quality of Life”.

Then, a second search using all identified keywords and index terms was undertaken across all included databases and outcome measures repositories. Thirdly, the reference list of all identified reports and articles was searched for additional studies. The electronic search was completed by hand search of articles cited in thematically relevant reviews and by sources provided by the steering committee and key personnel.

All articles were imported into a bibliographic software and, after removing duplicates, four reviewers independently screened the titles and abstracts and selected the studies to be included in this review. All the articles selected by at least two of the reviewers were retrieved for full text evaluation. The Population, Intervention, Comparison/ Control, Outcome, and Study design (PICOS) framework was used according to these search strategies:

Medline via Pubmed

#1: "interdisciplinary studies"[MeSH Terms] OR ("interdisciplinary"[All Fields] AND "studies"[All Fields]) OR "interdisciplinary studies"[All Fields]

#2: “multidisciplinary"[All Fields]
#3: #1 OR #2
#4: "approach"[All Fields]
#5: "conference"[All Fields]
#6: "team"[All Fields]
#7: "meeting"[All Fields] OR "meeting's"[All Fields] OR "meetings"[All Fields] OR "meets"[All Fields]
#8: "care"[All Fields]
#9: "discussion"[All Fields]
#10: "board"[All Fields]
#11: "management"[All Fields] OR "disease management"[MeSH Terms] OR ("disease"[All Fields] AND "management"[All Fields]) OR "disease management"[All Fields])
#12: "decision making"[MeSH Terms] OR ("decision"[All Fields] AND "making"[All Fields]) OR "decision making"[All Fields]
#13: "case review meeting"[All Fields]
#14: "collaborative approach"[All Fields]
#15: OR #4 - #14
#16: #3 AND #15
#17: ‘medical care team’
#18: ‘interdisciplinary care team’
#19: ‘multidisciplinary care team’
#20: ‘interprofessional care team’
#21: ‘patient care team’ OR ‘patient care team’[MeSH Terms]
#22: ‘team’ OR ‘teams’ OR ‘teamwork’ OR ‘team work’ OR ‘team-work’
#23: ‘shared care’
#24: ‘collaborative care’
#25: ‘integrated care’
#26: ‘team-based care’ OR ‘team based care’
#27: interprofessional* OR ‘interprofessional collaboration’ OR ‘interprofessional collaborative practice’
#28: OR #17 - #27
#29: #16 OR #28
#30: "lupus erythematosus, systemic"[MeSH Terms] OR ("lupus"[All Fields] AND "erythematosus"[All Fields] AND "systemic"[All Fields]) OR "systemic lupus erythematosus"[All Fields] OR ("systemic"[All Fields] AND "lupus"[All Fields] AND "erythematosus"[All Fields]
#31: #29 AND #30

Embase

#1: interdisciplinary
#2: 'multidisciplinary'
#3: #1 AND #2
#4: approach
#5: conference
#6: team
#7: care
#8: discussion
#9: meet OR meeting OR meetings

Journal of
Clinical Medicine

#10: board
#11: 'management' OR 'disease management'
#12: 'decision making'
#13: 'collaborative care team'
#14: OR #4 - #13
#15: #3 AND #14
#16: 'multidisciplinary care'/exp
#17: 'multidisciplinary care'
#18: 'multidisciplinary approach'/exp OR 'multidisciplinary approach'
#19: 'multi-disciplinary team'/exp OR 'multi-disciplinary team' OR 'multidisciplinary team'/exp OR 'multidisciplinary team'
#20: 'interdisciplinary care'/exp OR 'interdisciplinary care'
#21: 'interdisciplinary team'/exp OR 'interdisciplinary team'
#22: 'team based care'/exp OR 'team based care'
#23: 'interprofessional collaboration'/exp OR 'interprofessional collaboration'
#24: 'care, continuity of'/exp OR 'care, continuity of' OR 'continuity of care'/exp OR 'continuity of care' OR 'continuity of patient care'/exp OR 'continuity of patient care'
#25: 'episode of care'/exp OR 'episode of care'
#26: 'patient care'/exp OR 'patient care' OR 'patient care management'/exp OR 'patient care management' OR 'patient care team'/exp OR 'patient care team'
#27: 'patient centered care'/exp OR 'patient centered care' OR 'patient-centered care'/exp OR 'patient-centered care'
#28: 'patient helper'/exp OR 'patient helper'
#29: 'patient management'/exp OR 'patient management'
#30: 'patient navigation'/exp OR 'patient navigation'
#31: 'shared care'/exp OR 'shared care'
#32: 'collaborative care'/exp OR 'collaborative care'
#33: 'collective decision'/exp OR 'collective decision'
#34: 'team work'/exp OR 'team work' OR 'teamwork'/exp OR 'teamwork'
#35: 'integrated care'/exp OR 'integrated care'
#36: OR #16 - #35
#37: #15 OR #36
#38: 'lupus erythematosus'/exp OR 'lupus erythematosus' OR 'lupus.tw' OR 'sle.tw'
#39: #37 AND #38

Cochrane
#1: interdisciplinary
#2: 'multidisciplinary'
#3: #1 OR #2
#4: approach
#5: conference
#6: team
#7: care
#8: discussion
#9: meet OR meeting OR meetings

#10: board
#11: ′management'
#12: 'disease management'
#13: 'decision making'
#14: 'collaborative care team'
#15: OR #4 - #14
#16: #3 AND #15
#17: 'multidisciplinary care' OR 'multidisciplinary care'
#18: 'multidisciplinary approach' OR 'multidisciplinary approach'
#19: 'multi-disciplinary team' OR 'multi-disciplinary team' OR 'multidisciplinary team' OR 'multidisciplinary team'
#20: 'interdisciplinary care' OR 'interdisciplinary care'
#21: 'interdisciplinary team' OR 'interdisciplinary team'
#22: 'team based care' OR 'team based care'
#23: 'interprofessional collaboration' OR 'interprofessional collaboration'
#24: 'care, continuity of' OR 'care, continuity of' OR 'continuity of care' OR 'continuity of care' OR 'continuity of patient care' OR 'continuity of patient care'
#25: 'episode of care' OR 'episode of care'
#26: 'patient care' OR 'patient care'
#27: 'patient care management' OR 'patient care management'
#28: 'patient care team' OR 'patient care team'
#29: 'patient centered care' OR 'patient centered care'
#30: 'patient helper' OR 'patient helper'
#31: 'patient management' OR 'patient management'
#32: 'patient navigation' OR 'patient navigation'
#33: 'patient-centered care' OR 'patient-centered care'
#34: 'shared care' OR 'shared care'
#35: 'collaborative care' OR 'collaborative care'
#36: 'collective decision' OR 'collective decision'
#37: 'team work' OR 'team work' OR 'teamwork' OR 'teamwork'
#38: 'integrated care' OR 'integrated care'
#39: OR #17 - #38
#40:'lupus erythematosus' OR 'lupus erythematosus' OR 'lupus' OR 'sle'
#41: #39 AND #40

Cinahl
#1: interdisciplinary
#2: multidisciplinary
#3: #1 AND #2
#4: approach
#5: conference
#6: team
#7: care
#8: discussion
#9: meeting

Journal of
Clinical Medicine

MDPI

#10: board
#11: management OR disease management
#12: decision making
#13: collaborative approach
#14: OR #4 - #13
#15: #3 AND #14
#16: collaborative care
#17: team work
#18: shared care
#19: patient care team
#20: interprofessional care team
#21: multidisciplinary care team
#22: interdisciplinary care team
#23: medical care team
#24: integrated care
#25: OR #16 - #24
#26: #15 OR #25
#27: systemic lupus erythematosus OR lupus OR SLE
#28: #26 AND #27

The type of studies included were systematic literature reviews, meta-analyses, RCTs, controlled trials, non-controlled trials, diagnostic accuracy studies, cohort studies, cross-sectional studies, case-control studies and case series (> 5 patients). Only English and Italian language articles were included, while other languages and other study designs (narrative review, case reports <5 patients and meeting abstracts) were excluded. Studies were included if they addressed the outcome of SLE and fulfilled the predefined requirements. In case of disagreement between the reviewers, a further author was consulted to achieve a consensus. Selected articles were reviewed independently by four reviewers and all data were extracted using an extraction form designed to respond to primary and secondary objectives of the review. The following data were extracted and imported into a RedCap extraction form [1,2]: title of publication, study design, year of publication, subjects baseline demographics, sample size, country where study was conducted, ethnicity, baseline characteristics including type of intervention, outcome measures.

Bias was assessed according to the National Heart, Lung and Blood Institute risk-of-bias tool for any type of study [3]. Two reviewers rated each domain of the included studies as having a low, high or unclear risk of bias.

References

1 Harris PA, Taylor R, Thielke R, et al. Research electronic data capture (REDCap)--a metadata-driven methodology and workflow process for providing translational research informatics support. J Biomed Inform 2009;42:377–81. doi:10.1016/j.jbi.2008.08.010

Journal of
Clinical Medicine

2 Harris PA, Taylor R, Minor BL, et al. The REDCap consortium: Building an international community of software platform partners. J Biomed Inform 2019;95:103208. doi:10.1016/j.jbi.2019.103208

3 Study Quality Assessment Tools | NHLBI, NIH. https://www.nhlbi.nih.gov/health-topics/study-quality-assessment-tools (accessed 20 Jan 2023).
